# COLORECTAL CANCER: GLOBAL AND BRAZILIAN PERSPECTIVES, PREVENTION, AND THE IMPACT OF THE BLUE MARCH CAMPAIGN

**DOI:** 10.1590/S0004-2803.24612025-000

**Published:** 2025-04-04

**Authors:** Marcelo AVERBACH, Eduarda Nassar TEBET, Eduardo Guimarães Hourneaux de MOURA

**Affiliations:** 1 Livre Docente pela Faculdade de Medicina da USP. Docente do Instituto Sírio Libanês de Ensino e Pesquisa. Coordenador da Campanha Março Azul. Presidente da ONG Zoé, Brasil.; 2 Preceptora da residência de gastroenterologia Hospital Regional Rosa Pedrossian- MS. Coordenadora do Núcleo de Ações Sociais da Sociedade Brasileira de Endoscopia Digestiva, Brasil.; 3 Professor Livre-Docente do Depto de Gastroenterologia da FMUSP. Coordenador do Serviço de Endoscopia do Aparelho Digestivo do Hospital Vila Nova Star. Presidente da SOBED 2025-2026, Brasil.

Colorectal cancer (CRC) is one of the most prevalent malignancies worldwide, affecting both men and women. It primarily involves the colon or rectum and is associated with significant morbidity and mortality. Understanding its global frequency, the particularities in Brazil, and the role of prevention campaigns such as the Blue March is crucial for addressing this significant public health issue.

Frequency of Colorectal Cancer Globally, CRC is the third most commonly diagnosed cancer and the second leading cause of cancer-related deaths^1^. According to the Global Cancer Observatory (GLOBOCAN 2020), over 1.9 million new cases and 935,000 deaths were reported worldwide that year. Developed regions, such as North America, Europe, and parts of Asia, report higher incidence rates, partly due to lifestyle factors and better screening programs that increase case detection. However, the disease is also rising in low- and middle-income countries due to aging populations, urbanization, and changes in dietary habits^2^.

In Brazil, CRC is the second most common cancer among men and women, excluding non-melanoma skin cancers. The Brazilian National Cancer Institute (INCA) estimated approximately 45,000 new cases annually between 2023 and 2025^3^. The incidence and mortality rates vary significantly across Brazilian regions, with higher rates observed in the South and Southeast, likely due to better diagnostic facilities and lifestyle patterns.

## Risk factors several factors contribute to CRC’s prevalence:

- Lifestyle habits: diets rich in processed foods and red meat, combined with low physical activity levels, increase risk^4^.
- Obesity: rising obesity rates are a significant concern.
- Healthcare disparities: limi^­^ted access to screening in some regions leads to delayed diagnoses.

Prevention and early detection prevention are paramount in reducing CRC incidence and mortality. Strategies include:

## Primary prevention

### 1. Lifestyle modifications:

- Adopting a diet rich in fruits, vegetables, and whole grains while reducing red and processed meat consumption.
- Regular physical activity and maintaining a healthy weight.
- Limiting alcohol intake and avoiding tobacco use.

### 2. Public education:

- Raising awareness about risk factors and symptoms, such as rectal bleeding, abdominal pain, and changes in bowel habits.

## Secondary prevention

### 1. Screening programs:

- Regular screening is vital, as it detects precancerous polyps and early-stage cancers.
- Recommended methods include fecal immunochemical tests (FIT), fecal occult blood tests (FOBT), and colonoscopy.
- In Brazil, colonoscopy remains underutilized due to resource limitations.

### 2. High-risk populations:

- Individuals with a family history of CRC or genetic predispositions (e.g., Lynch syndrome) require tailored surveillance.

The Blue March (Março Azul) campaign, inspired by international efforts like the United States’ Colorectal Cancer Awareness Month, plays a pivotal role in Brazil’s fight against CRC. It aims to:

## Goals

### 1. Raise awareness:

- Educate the public about CRC risk factors, symptoms, and the importance of screening.

### 2. Promote screening:

- Advocate for regular screening among individuals aged 45 and above or earlier for those with a family history.

### 3. Reduce stigma:

- Address cultural barriers surrounding colonoscopy and other diagnostic tests.

## Activities

- Public lectures, media campaigns, and social media outreach to disseminate information.
- Illuminating public landmarks in blue to symbolize CRC awareness.
- Organization of task forces in selected cities to conduct screening through fecal occult blood tests, followed by colonoscopy for positive cases. These initiatives have already been carried out in the cities of Belterra (PA), Penedo (AL), Piranhas (AL), Cairu (BA), and Óbidos (PA)^5^.
- This year, the task force organized by the Brazilian Society for Digestive Endoscopy (SOBED) and the Brazilian Society of Coloproctology (SBCP) will take place in Goiás (GO), a city with a population of 24,071. The initiative aims to offer approximately 8,000 FIT tests and perform an estimated 500 to 600 colonoscopies.

## Impact

The Blue March has significantly increased awareness and participation in CRC prevention efforts. However, disparities in healthcare access and regional differences in campaign reach remain challenges.

In summary, colorectal cancer poses a significant global and national health challenge. While Brazil has made strides in prevention and awareness through initiatives like the Blue March campaign, continued efforts are needed to address regional disparities, improve access to screening, and promote healthy lifestyles. By prioritizing early detection and public education, the burden of CRC can be reduced, saving countless lives.
